# scACCorDiON: a clustering approach for explainable patient level cell–cell communication graph analysis

**DOI:** 10.1093/bioinformatics/btaf288

**Published:** 2025-05-06

**Authors:** James S Nagai, Tiago Maié, Michael T Schaub, Ivan G Costa

**Affiliations:** Institute for Computational Genomics, RWTH Aachen Medical Faculty, Aachen 52074, Germany; Institute for Computational Genomics, RWTH Aachen Medical Faculty, Aachen 52074, Germany; Department of Computational Science, RWTH Aachen University, Aachen 52074, Germany; Institute for Computational Genomics, RWTH Aachen Medical Faculty, Aachen 52074, Germany

## Abstract

**Motivation:**

Combining single-cell sequencing with ligand–receptor (LR) analysis paves the way for the characterization of cell communication events in complex tissues. In particular, directed weighted graphs naturally represent cell–cell communication events. However, current computational methods cannot yet analyze sample-specific cell–cell communication events, as measured in single-cell data produced in large patient cohorts. Cohort-based cell–cell communication analysis presents many challenges, such as the nonlinear nature of cell–cell communication and the high variability given by the patient-specific single-cell RNAseq datasets.

**Results:**

Here, we present scACCorDiON (single-cell Analysis of Cell–Cell Communication in Disease clusters using Optimal transport in Directed Networks), an optimal transport algorithm exploring node distances on the Markov Chain as the ground metric between directed weighted graphs. Benchmarking indicates that scACCorDiON performs a better clustering of samples according to their disease status than competing methods that use undirected graphs. We provide a case study of pancreas adenocarcinoma, where scACCorDion detects a sub-cluster of disease samples associated with changes in the tumor microenvironment. Our study case corroborates that clusters provide a robust and explainable representation of cell–cell communication events and that the expression of detected LR pairs is predictive of pancreatic cancer survival.

**Availability and implementation:**

The code of scACCorDiON is available at https://scaccordion.readthedocs.io/en/latest/. and https://doi.org/10.5281/zenodo.15267648. The survival analysis package can be found at https://github.com/CostaLab/scACCorDiON.su.

## 1 Introduction

Single-cell RNA sequencing (scRNA-seq) enables the characterization of cellular processes at unprecedented resolution. Specifically, it allows the study of cell–cell communication (CCC) via the expression patterns of cognate ligand–receptor (LR) pairs across cells detected via scRNA-seq (Armingol *et al.* 2021, Dimitrov *et al.* 2022). As sequencing costs have been reduced by the rapid improvement of single-cell sequencing protocols, it has become possible to create scRNA-seq datasets for large patient cohorts (CZI Single-Cell Biology *et al.* 2023). Such datasets, which contain patients under different conditions, have the potential to improve understanding of how cell communication changes in various biological settings. However, for a sample-level analysis of such large-scale scRNA-seq patient data, efficient computational approaches are needed (Flores *et al.* 2023, Joodaki *et al.* 2024).

There are now hundreds of computational methods for LR-based communication analysis (Armingol *et al.* 2024). These tools mainly focus on inferring LR pairs within a *single* biological condition. A yet poorly studied aspect is to characterize changes in cell communication in *multiple* biological conditions, such as disease versus control (Nagai *et al.* 2021) or over cell differentiation (Li *et al.* 2022). To this date, only a few computational methods for CCC—Tensor2Cell and MultiNicheNet—have considered data from multiple samples (patients). MultiNicheNet (Browaeys *et al.* 2023) builds upon NicheNet (Browaeys *et al.* 2020), considering both extra-cellular and intra-cellular signaling in CCC. To consider multiple samples, MultiNicheNet obtains pseudo-bulk representations, where cells are bulked for each cell type and sample, and uses a differential expression approach [edgeR, Robinson *et al.* (2010)] to perform a multiconditional differential communication analysis. However, MultiNicheNet is a supervised algorithm that requires the group of samples to be defined prior and thus does not allow for the identification of unknown groups of patients with distinct CCC programs. TensorCell2Cell (Armingol *et al.* 2022) uses tensor component analysis to detect latent factors explaining changes in CCC associated with sample-level scRNA-seq data. The factors can detect patterns (CCC events) related to individual samples. Similar to MultiNicheNet, Tensor2Cell does not provide any approach for finding unknown groups of samples defined by distinct CCCs.

This work explores CCC across multiple patients using directed weighted graph representations. In this representation, cell types are nodes; directed edges represent a communication event connecting a source cell (expressing a ligand) to a target cell (expressing a cognate receptor) (Nagai *et al.* 2021). The combined expression of LR molecules represents the strength of these directed edges (or edge weight). Using a graph representation enables us to exploit a wide variety of graph algorithms, such as pagerank (Page *et al.* 1998), to detect latent cell–cell communication events leading to fibrosis (Leimkühler *et al.* 2021, Jansen *et al.* 2022). Within the sample-level cell–cell communication context, a common challenge in the sample-level analysis is clustering the samples according to disease stages. When using a graph-based representation, this corresponds to clustering a set of graphs according to their similarity, which is a computationally challenging task. This problem has previously been tackled with graph-based optimal transport (OT) approaches, which (implicitly) utilize spectral properties of graphs (Maretic *et al.* 2019) or node distances (Xu *et al.* 2019, Scholkemper *et al.* 2024). However, these approaches are designed for undirected graphs and would miss important information regarding the directionality of LR interactions.

**Figure 1.**
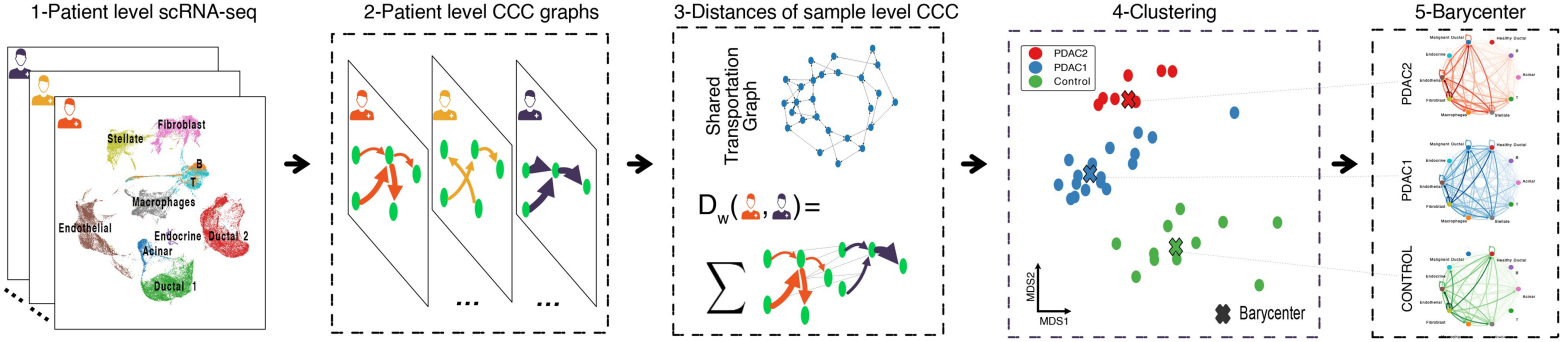
Overview of scACCorDiON: scACCorDiON receives a scRNA-seq cohort experiment as input. In the first step (1 and 2), LR analysis is performed, and the cell–cell communication graphs for every sample (patient) are recovered. Next (step 3), we use graph-based optimal transport, which considers directions and weights of cell–cell interactions, to measure the distance between all pairs of samples. In the third step (4 and 5), this distance is used as input for *k*-medoids or *k*-barycenter algorithms that find groups of CCC graphs and produce representative CCC graphs for different patient conditions, and for dimension reduction algorithms to create low-dimensional data visualizations. In summary, scACCorDiON enables the analysis of patient cohorts at a CCC graph level and allows for a quantitative comparison of the changes in CCC graphs using optimal transport. Moreover, Barycenters/Medoids are a proxy to facilitate the explainability of the produced results.

## 2 Approach

We propose scACCorDiON (single-cell Analysis of Cell–Cell Communication in Disease clusters using OT in directed Networks). scACCorDiON represents the CCC data of each sample as a directed weighted graph (DWG) and uses the Wasserstein distance (Bonneel *et al.* 2011) between CCC graphs to derive patient-patient distances (Fig. 1). For this, we assume that the probability masses to be transported via OT correspond to the directed cell pair interaction strength (LR expression values). scACCorDiON adopts a balanced OT approach, i.e. it considers that the “mass” of cell–cell communication signals is conserved between patients and that the same amount of cell–cell communication is present in both normal and disease samples. To model this, we lift each CCC graph to a line graph, where a node represents a directed interaction, and its mass (or weight) represents the LR expression values of this interaction. scACCorDiON encodes interaction information in two ways. First, it uses a cost function for OT, the Hitting Time Distance (HTD) (Boyd *et al.* 2021), a distance obtained by considering the directed graph as a Markov chain. Moreover, by working in a line graph, the masses to be transported are related to a directed cell pair interaction.

We use two clustering approaches with the estimated distance matrices: a k-medoid algorithm (Lloyd 1982) and a barycenter algorithm. For the latter, we take advantage of the fact that OT enables us to estimate the barycenters of a set of CCC networks (Cuturi and Doucet 2014). These barycenters can be used as “centroid” values within an expectation-maximization clustering algorithm denoted *k*-barycenters. Also, barycenters can be used together with transport maps for interpretation, i.e. delineating cell communication events that change between groups of samples.

We benchmarked scACCorDiON with the undirected graph OT (GOT) (Maretic *et al.* 2019) and baseline approaches: tabular representation of the data ignoring graph structures, and a simple OT approach exploring the correlation of nodes in undirected CCC graphs. The benchmark tested how well methods can recover known disease labels of samples across seven large scRNA-seq cohorts with up to 126 samples and up to a million cells. MultiNicheNet or Tensor2Cell could not be evaluated as neither method allowed the unsupervised analysis of samples. We assess how well clustering methods can recover the known disease labels of the samples.

Afterward, we explore the clustering results and transport maps to characterize CCC events on a pancreas adenocarcinoma scRNA-seq data (Peng *et al.* 2019). scACCorDiON can detect novel uncharacterized sub-groups of disease samples and related LR pairs. Moreover, we use external data from the The Cancer Genome Atlas (TCGA), to show that sub-cluster specific LR interactions can predict pancreas adenocarcinoma patients’ survival, supporting the translational use of scACCorDiON. Finally, we contrasted results from scACCorDiON latent spaces derived from Tensor-cell2cell, showing both approaches’ complementarity.

## 3 Materials and methods

### 3.1 scACCorDiON

scACCorDiON is an OT-based framework for directed weighted graph metric learning and clustering. The input to scACCorDiON is a set of directed weighted CCC graph $\left\{G^{1},\ldots ,G^{p}\right\}$ containing *p* graphs. We assume that each of these *p* graphs has been obtained from a LR analysis method (Nagai *et al.* 2021, 2024), applied to a scRNA-seq dataset containing multiple samples (e.g. a cohort). The CCC graph of sample *k* is then defined as $G^{k}=(V^{k},E^{k},w^{k})$, where a node $v\in V^{k}$ represents a cell type, and a directed edge $e\in E^{k}$ connects a pair of cell types when these cells are predicted to be communicating through a ligand (source cell) and receptor (target cell) pair. The weights of the edges $w^{k}$ are related to the amount of communication between the source and target cell, e.g. $w^{k}(e)$ is the sum of all LR expressions (LRScore). Note that in our problem setup, nodes can be identified across all graphs for a given scRNA-seq dataset, i.e. all samples *k* have the same cell types and thus the same node-set $V^{k}=V$.

#### 3.1.1 Metric learning from directed weighted graphs

scACCorDiON uses OT to obtain a metric between patients’ CCC graphs (Bonneel *et al.* 2011). More specifically, we hypothesize that the edge weights of each graph are a specific realization of a signal supported on the same underlying graph structure, i.e. every CCC graph has distinct signals related to the LR expression between cell pairs, but they all share the same topology. Therefore, the directed weighted graph OT (DW-OT) problem we consider here consists of finding an OT map between the CCC graph edge signals with respect to an edge-to-edge cost (distance) matrix C. For this, we proceed in two steps. First, we define a shared topology line graph, which only considers directed edges present in at least one sample and that weights these edges by their frequency in the data (Supplementary Fig. S1). This graph is used to compute the OT cost function using an approach based on Markov chain theory (Boyd *et al.* 2021). Second, we treat each sample’s edge weights as a signal distribution (LR expression) on this line graph and use OT to transport two such distributions.

### 3.2 Shared topology graph (STG)

We build a *directed line graph* L=(V,E,W), whose vertex set V contains each possible edge (j,k) contained in one of the sample graphs $G^{i}$. The edge set E of the linegraph is defined as follows: an edge $e^{\prime }=(u^{\prime },v^{\prime })\in E$ exists if the target of $u^{\prime }$ is the source of $v^{\prime }$, i.e. the target node of edge $u^{\prime }$ in the original graph, is the source node of edge $v^{\prime }$ in the original graph. An schematic illustrating the STG construction is available in Supplementary Fig. S1. Note that this line graph’s structure essentially encodes the union of interactions of all CCC graphs. We denote this graph as the “shared topology graph” (STG). Finally, we defined the weight $w_{u^{\prime }v^{\prime }}$ of edge $\left(u^{\prime },v^{\prime }\right)$ as the proportion of graphs containing both edges $u^{\prime }$ to $v^{\prime }$. This makes transport of masses between common edges (in the original graphs) more likely than transport between rare edges (in the original graphs). As our line graph can have unconnected components, we add to the STG a low-rank regularization term, as popularized within the context of the well-known PageRank algorithm (Page *et al.* 1998, Gleich 2015), to obtain a well-posed problem. Specifically, this guarantees the global reachability of all nodes in the STG, which is required to compute the distance between nodes in a graph. See Supplementary Fig. S2 for an example of an evaluation of the effect of this parameter. Using the STG, we can represent each sample as a signal distribution on the nodes of the STG, which we can compare via OT. However, for the computation of OT, we also need to define a distance matrix for the nodes on the STG, which specifies the cost of moving a signal from one node to another node in the STG.

### 3.3 Hitting time distance (HTD)

Here, we consider the Hitting Time Distance [HTD (Boyd *et al.* 2021)], which is a metric that can be applied to *directed* weighted graphs. To derive the HTD, we consider a discrete-time Markov chain ${\left(X_{t}\right)}_{t\geq 0}$ defined over the vertices V={1,…,N} of a strongly connected graph. We assume the chain has a starting distribution λ and an irreducible transition matrix $P=D^{-1}A$, where *A* is the adjacency matrix of the shared topology graph and D=diag(A1). The Markov chain can then be described according to the state transition probabilities:

(1) $$P(X_{0}=i)=\lambda_{i}\;\text{and}\;P(X_{t+1}=j|X_{t}=i)=P_{i,j}.$$

Let $\pi \in R^{N}$ be the invariant distribution of the chain, i.e. πP=π. For a starting point distributed according to λ, the *hitting time* of a vertex i∈V is the random variable $\tau_{i}=\inf\{t\geq 1:X_{t}=i\}$. Following Boyd *et al.* (2021), we define the probability that starting in a node *i*, the hitting time of *j* is less than the time it takes to return back to *i* by $Q_{i,j}:=P(\tau_{j}\leq \tau_{i}|X_{0}=i)$. Based on the matrix $Q=[Q_{i,j}]$ a normalized hitting time matrix **T** can be defined in terms of its entries

(2) $$T_{i,j}=\{\begin{array}{cc} \frac{\pi_{i}^{1/2}}{\pi_{j}^{1/2}}Q_{i,j} & i\neq j,\\ 1 & \text{otherwise}, \end{array}$$

If **P** is an irreducible stochastic matrix, i.e. the underlying graph is strongly connected, the Hitting Time Distance Matrix can be obtained by:

(3) $$C_{\text{HTD}}(i,j)=-\text{log}(T_{i,j})$$

This distance can now be used as a cost matrix C for an OT problem that considers the movement of signal masses on the STG for different samples.

### 3.4 Computing a graph-based CCC distance

To set up our OT-based distance, let us collect the edge weights of each CCC graph in a matrix $P\in R^{p\times E}$, where *E* is the size of the union of the edge sets of all graphs (samples). Stated differently, *E* corresponds to the number of nodes in the line graph. Hence, the columns of P are indexed by the (directed) edges and rows by the samples/graphs, i.e. the row $P_{k,:}$ describes the edge-weights $w^{k}$ of the *k*th graph $G^{k}$, which is appropriately zero-padded, in case $G^{k}$ does not contain certain edges which are present in other graphs.

The optimal transport map $\Gamma^{*}\in R^{E\times E}$ for two probability distributions defined on the nodes of the line graph as induced by the two CCC graphs $G^{k}$ and $G^{l}$ can now be computed as

(4) $$\begin{array}{c} \Gamma^{*}=\arg\underset{\Gamma \in S}{\min}\;{\langle \Gamma ,C\rangle }_{F},\\ w\mathrm{here}S=\left\{\Gamma |\Gamma 1=P_{:,k},\Gamma^{T}1=P_{:,l},\Gamma_{ij}\geq 0\right\}, \end{array}$$

and the associated (induced) Wasserstein distance between the two CCC samples is:

(5) $$d_{W}(G^{k},G^{l})=\underset{\Gamma \in S}{\min}{\langle \Gamma ,C\rangle }_{F}={\langle \Gamma^{*},C\rangle }_{F}$$

**Remark.** scACCorDiON uses a balanced optimal transport, which assumes a mass conservation assumption. In our benchmarking, we also consider the unbalanced formulation described in the Supplementary Methods.

### 3.5 Clustering patient’s networks

scACCorDiON uses the metric $d_{W}$ [Equation (5)] to perform clustering of CCC graphs. One approach for this is a *K*-medoids partitioning algorithm (Rdusseeun and Kaufman 1987, Schubert and Rousseeuw 2019, 2021), which only requires a distance matrix and detects samples (medoids) as representative of clusters. scACCorDiON leverages that we can also compute barycenters for distributions of (directed, weighted) graphs via the Wasserstein optimal transport framework (Cuturi and Doucet 2014).

#### 3.5.1 K-barycenters clustering

A Wasserstein barycenter of a set of graphs $G=\{G^{1},\ldots ,G^{p}\}$ can be defined as:

(6) $$\mathrm{barycenter}(G)=\arg\underset{\mu }{\min}\frac{1}{p}\sum_{i=1}^{p}d_{W}(G^{i},\mu (i)).$$

Time-efficient solutions to this problem can be obtained by using a dissimilarity-based loss function (Sinkhorn) of the optimal transport algorithm (Cuturi and Doucet 2014). In addition, we use barycenters to define an expectation-maximization-based clustering algorithm, where barycenters represent the “centroids” and we use the Wasserstein distance [Equation (5)] between graphs and barycenters. Given *k* as the number of desired clusters, *Y* be an indicator variable, where $y_{i}\in \{1,\ldots k\}$ indicates the cluster of $G^{i}$, and $\left\{\mu^{1},\ldots ,\mu^{k}\right\}$ indicates the set of barycenters, this leads to clustering algorithm (Algorithm 1).

Algorithm 1 K-Barycenters

**  Input:** CCC graph’s $\left\{G^{1},\ldots ,G^{p}\right\}$ and number of clusters k∈N

**  Output:**  $y=(y_{1},\ldots ,y_{p})$ where $y_{i}\leq k$

1: ** **Initialize barycenters ($\mu_{1}$,…,$\mu_{k}$)

2: ** repeat**

3: **  **Expectation Step:

4: **  **$\;y_{i}=\mathrm{argmi}n_{j=1,\ldots ,k}d_{w}(G^{i},\mu_{j}),\forall i\in p$

5: **  **Maximization Step:

6: **  **$\mu_{j}$ = barycenter($\left\{G|G^{i}\in G,y_{i}=j\right\}$)

7: ** until** Barycenter does not change

To avoid local maxima due to the random initialization, we repeat the optimization process 100 times and select the solution with the lowest average Wasserstein loss per cluster. Moreover, we use a seeding process to pick the initial barycenters based on selecting CCC graphs that maximize the cluster-to-cluster distance as described in Arthur *et al.* (2007).

### 3.6 Benchmarking

For benchmarking, we have collected seven publicly available disease scRNA-seq cohorts, from which samples were annotated with their disease status. We obtained pre-processed, integrated, clustered, and annotated objects for all datasets from (CZI Cell Science Program *et al.* 2025). An exception is the pancreas adenocarcinoma datasets, which were pre-processed as described in Joodaki *et al.* (2024).

The LR inference was performed with CellPhoneDB (Efremova *et al.* 2020) implemented in the LIANA (Dimitrov *et al.* 2022) framework by only considering cells in a patient sample. The parameter related to the minimum expression proportion for the ligands/receptors is set to exp _prop=0.15, and highly significant interactions were considered *P*-value ≤ 0.01. A description of the dataset’s main features is provided in Table 1. While scACCorDiON can be used with any LR inference algorithm, we choose CellPhoneDB due to its widespread use in the literature. We also note that CellPhoneDB is the best-performing tool in the recent single-cell benchmark (Luecken *et al.* 2024). CCC graphs were generated using CrossTalkeR (Nagai *et al.* 2021).

**Table 1.** Main features of datasets used in the benchmark, including the number of cell types (Cells), the average number of directed cell–cell interactions (CCI) detected with LR analysis, number of individuals/samples, number of cells, and number of sample labels.

Data/study	Cells	CCIs	Samp.	No. of cells	Label	References
Pancreas Adenocarcinoma	10	75	35	57.530	2	Peng *et al.* (2019)
COVID	18	245	130	647.366	4	Stephenson *et al.* (2021)
Myocardial infarction	33	871	23	132.888	3	Kuppe *et al.* (2022)
Breast cancer	10	94	126	714.331	2	Kumar *et al.* (2023)
Kidney AKI	13	142	36	76.020	3	Lake *et al.* (2023)
Lung atlas	12	117	165	941.504	5	Sikkema *et al.* (2023)
RCC	40	1280	17	50.236	2	Zvirblyte *et al.* (2024)

We are unaware of another computational approach that can cluster samples by considering CCC information. However, we can contrast scACCorDiON with the following baseline approaches. First, we consider tabular representations (edge weight matrix P) of the data as input (Tabular). Due to the high dimensionality of P, we first perform a dimension reduction with Principal Component Analysis (PCA). We compute the correlation distance on the matrix P as an OT baseline method and cost function for the previously described OT framework. This approach is denoted CORR-OT. Note that the last approach does not consider the graph’s directions. We also included an undirected graph optimal transport method in our benchmark, GOT (Maretic *et al.* 2019). GOT receives a single graph as input for every patient, where two cell types are connected with the cell pairs detected in one of the directions. The average LR scores (one for each direction) give the edge weights.

For distance metrics obtained by evaluated methods (Tabular, GOT, CORR-OT and DW-OT), we run *k*-barycenters, *k*-medoids and the community detection algorithm Leiden (Traag *et al.* 2019). The last algorithm is chosen based on its widespread use in scRNA-seq pipelines (Wolf *et al.* 2018). Note also that for Tabular, we use *k*-means algorithm as this is equivalent to a *k*-barycenter in an Euclidean space. Algorithms were run by varying the number of clusters from 2 to 7. The Adjusted Rand Index (Hubert and Arabie 1985) (ARI) and Rand Index (Rand) for the *k* equal to the number of class labels and *k* with maximum ARI value were computed for each clustering. Here, the disease labels are used as true classes. The Friedman-Nemenyi post-hoc test was used for every metric, clustering, and distance combination to statistically address the rank differences (Nemenyi 1963, Demšar 2006). For Leiden (Traag *et al.* 2019), we vary the resolution parameter from 0 and 1 with 0.01 steps, as this allows us to obtain distinct clusters. We refer to Supplementary Fig. S3 for an overview of the experimental design. To explore the interpretability of Tensor-cell2cell (Armingol *et al.* 2022), we also estimated tensor decomposition and contrasted results with the disease labels and the new clustering by scACCorDiON. Here, we followed the tutorial available in https://liana-py.readthedocs.io/en/latest/notebooks/liana_c2c.html. Elbow optimization was performed to select the optimal number of factors (8).

### 3.7 Robustness analysis

We also analyzed the robustness of scACCorDiON concerning the cell type annotation resolution. For this, we evaluated the clustering performance under two annotation levels (major cell types, and refined cell types/sub-states), provided in the myocardial infarction (MI) and the renal clear carcinoma (RCC) datasets. Moreover, the performance of under-sampling the number of cells in the PDAC dataset was also evaluated, i.e. we randomly removed up to 75% of cells and performed LR and scACCorDiON analysis. For each stratum, five random samples were generated.

### 3.8 LR survival analysis

As a form of independent validation, we evaluate if the top predicted LRs, i.e. related to cells relevant to PDAC subgroups, could function as predictors of survival in the PAAD (Pancreatic Adenocarcinoma) TCGA dataset (Raphael *et al.* 2017). Given a list of candidate LR pairs, we estimate LRScores on the bulk-RNA set data by computing the geometric mean of the LR pairs on the expression data. Finally, we use a Cox Proportional Hazards model (Andersen and Gill 1982) to compute the Hazard Ratios adjusted to the cancer stage covariate and the LRs. To this end, Stage III and IV were aggregated into Stage III+ due to the low number of samples. We compute the log-rank test to observe the direct relation of the LRs with overall survival.

## 4 Results

### 4.1 Benchmarking cell–cell communication graph clustering

We evaluated the performance of scACCorDiON and baseline competing methods using seven publicly available scRNA-seq cohort datasets. The datasets contain between 10 and 33 cell types, 20 and 165 samples, and 50 236 to 941 504 single cells. CCC graphs have an average interaction number between 75 and 142 (Table 1). scACCorDiON’s mainly consists of using the *k*-medoids and *k*-barycenter clustering algorithm with a Wasserstein distance considering both the direction and topology of graphs (DW-OT). In the evaluation, we include a baseline OT method (CORR-OT), which considers the signal directions but not the topology of the CCC graphs; as well undirected graph optimal transport algorithm GOT (Maretic *et al.* 2019).

To evaluate the impact of the clustering method, we also performed clustering for all methods with *k*-medoids and Leiden algorithm (Traag *et al.* 2019). All methods are evaluated with respect to their performance in the recovery of clusters related to the known class labels measured by the ARI (Hubert and Arabie 1985). Class labels indicate the individual’s health status: healthy versus diseased (or disease sub-type). ARI is measured for the number of *k* equal to the number of true labels or the maximum ARI after varying the number of *k* from 2 to 7 (or cluster resolution) for a given dataset and algorithm. The corresponding individual line plots are displayed in Supplementary Fig. S5. Additionally, we repeated the evaluation assay using the rand index (Rand 1971), considering the agreement of two partitions without any correction.

**Figure 2.**
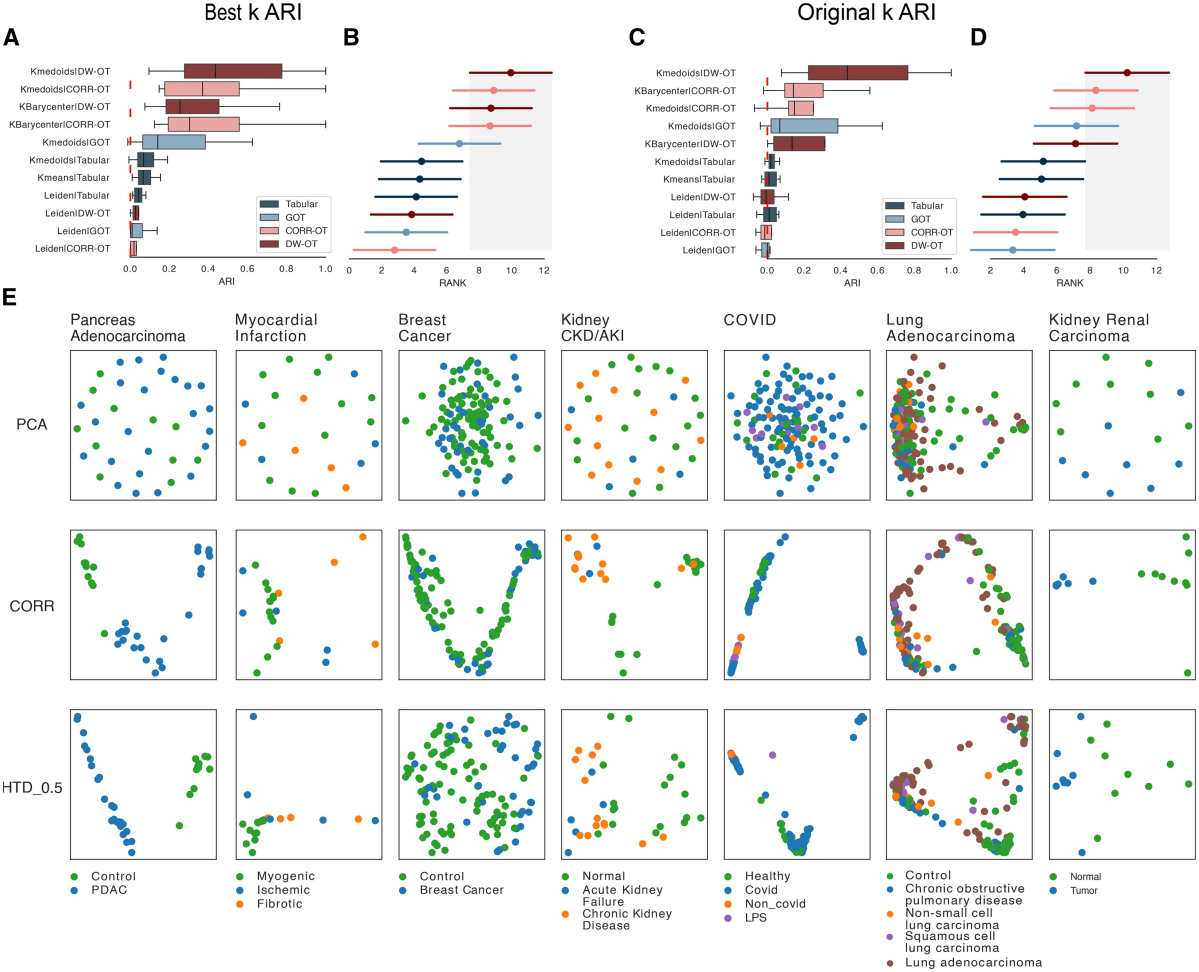
Clustering benchmark: (A) boxplots indicate the maximum ARI value distribution (*x*-axis) distribution for all evaluated methods over five scRNA-seq datasets. (B) Ranking values (mean and std) for each method and dataset regarding the maximum ARI value. The highest ranking indicates the highest ARI. The gray area indicates the 95% confidence interval of the Friedman and Nemenyi post-hoc test). Methods whose average values are not within the gray area have significantly lower rankings than the top-ranked methods. For both A and B, methods are ranked by average in decreasing order. C and D is the same as A and B for the ARI estimates, with the number of clusters equal to the number of original labels. (E) PHATE 2D embeddings of the distances matrices estimated by Tabular, CORR-OT, and DW-OT for all evaluated datasets. Colors correspond to the original labels.

The benchmark results are shown Fig. 2A–D, and Supplementary Table S1. We observe that DW-OT with k-medoids has the highest mean ARI for the maximum ARI evaluation (Fig. 2A). A Friedman-Neymeni test indicates that DW-OT with *k*-medoids has the highest ranking and significantly outperforms Tabular based baseline approaches and the use of Leiden clustering (Fig. 2B). DW-OT with k-medoids obtains the highest mean ARI for the number of clusters equal to the number of classes (Fig. 2C and D). A Friedman-Neymeni test indicates that also this *k*-medoids variant outperforms Tabular based baseline approaches and the use of Leiden clustering. Figure 2E (Supplementary Fig. S4C) shows embeddings (Moon *et al.* 2017) obtained from distances generated in this study. Regarding Rand Index, we observe similar rankings of methods and an average Rand index varying from 0.6 to 0.8 for DW-OT. These results underline the advantage of DW-OT with k-medoids, which is the only approach incorporating both directionality and connectivity of CCC graphs to cluster the samples.

We also evaluate how some of the methodological choices impact the results from scACCorDiON. First, we evaluate if the granularity of the cell type annotation impacts the overall results in the MI and RCC datasets, which provide two level of cell type annotation. Results (Supplementary Table S2) indicates that in both cases, DW-OT obtain best results at course annotation levels. This supports that cellular sub-state annotation is beneficial for cell–cell communication prediction. Another important question is the robustness of the results in relation to the quality of the scRNA-seq, i.e. recovery of cells per cluster. A cell down-sampling analysis in the PDAC data indicates that scACCorDiON only slightly deteriorates (0.12 ARI) when only 37.5% of the cells are kept.

One assumption of scACCorDiON is the fact that masses are conserved, i.e. the same level of cell–cell communication is present in distinct samples. To evaluate this, we contrast the performance of the balanced and unbalanced OT formulations (See Supplementary Material). An analysis of DW-OT with the balanced OT versus unbalanced versions, indicates no statistical difference between approaches. Nevertheless, the balanced DW-OT obtains the higher ARI (and RI) scores (Supplementary Fig. S6). This supports the feasibility of the hypothesis of mass conservation as explored by scACCorDiON.

### 4.2 scACCorDiON detects a Sub-cluster of pancreas adenocarcinoma

**Figure 3.**
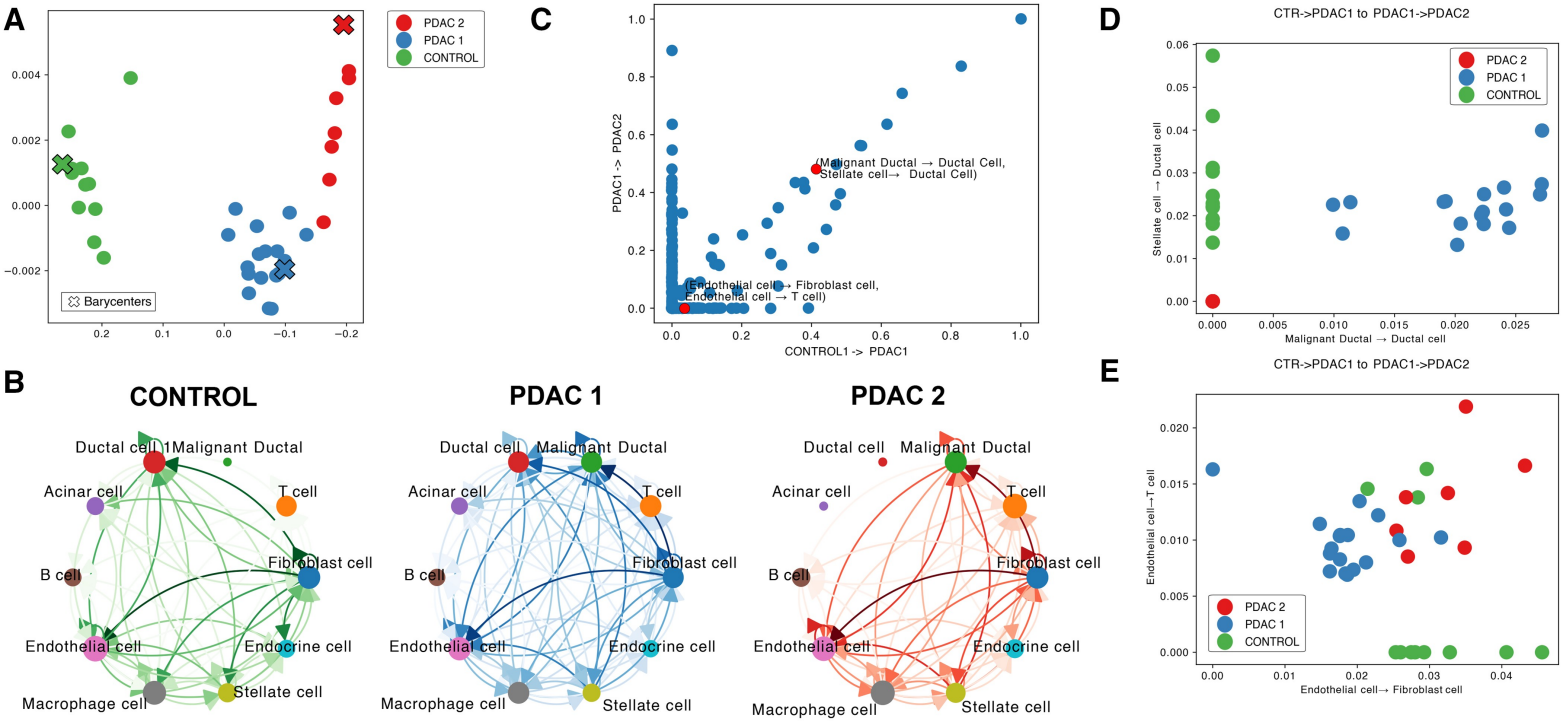
Sub-cluster analysis in pancreas adenocarcinoma. (A) MDS plot containing the clustering results on pancreas adenocarcinoma. One cluster contains all control samples (Control), and two clusters contain disease samples (PDAC 1 and 2). (B) Directed CCC graphs of barycenters of the detected sub-clusters. Edge thickness indicates the strength of the cell–cell interactions. Node sizes are placed accordingly to the node pagerank, and low representation edges were filtered to improve visualization. (C) Scatter plot with the transport map between the barycenter of the control and PDAC 1 (*y*-axis) and PDAC 1 and PDAC2 clusters (*y*-axis). Every dot shows the mass transported between two cell–cell pairs in the comparisons. (D and E) Scatter plot with signals associated with selected cell–cell pairs for all the samples shown in (A). Colors correspond to the sample cluster.

To evaluate the power of scACCorDiON in the detection of novel sub-clusters, we perform a Silhouette analysis (Rousseeuw 1987) to identify datasets with a higher number of clusters than true labels (Supplementary Fig. S5). Interestingly, we observe that for the pancreas adenocarcinoma (PDAC) data, scACCorDiON predicts a sub-cluster associated with controls and two sub-groups associated with PDAC samples (Fig. 3A). As displayed in Fig. 3B, PDAC 1 has overall increased communication, particularly interactions related to ductal, malignant ductal, and fibroblast cells. PDAC 2 demonstrates a loss of communication regarding Acinar and Ductal cells, while we observe an increase in communication related to Malignant Ductal, B, and Endocrine cells. The prominent signal related to the Malignant Ductal Cells indicates that PDAC 2 clusters are, possibly, linked to more advanced disease stages than the PDAC 1 cluster (Peng *et al.* 2019).

To understand CCC events related to transitions from control to early disease (Control –> PDAC 1). Between mild and advanced disease (PDAC 1->PDAC 2), we contrast the transport maps (Γ) between the barycenters of these pairs of groups (Fig. 3C). We observe that pairs of CCC interactions with high transport masses discriminate well the detected groups (Fig. 3D and E).

We next make use of the LR analysis from CrossTalkeR (Nagai *et al.* 2021) to further investigate the interactions associated with the communication between Malignant Ductal cells and Ductal cells (Supplementary Fig. S8). We observed high expression in ERBB and EGFR receptors’ interactions among the top LR pairs. These receptors were previously assigned to be related to pancreatic intraepithelial neoplasia (PanIN) (Ghasemi *et al.* 2014, Meyers *et al.* 2020), described as a precursor stage of Pancreas Adenocarcinoma. Moreover, ligands secreted by Malignant Ductal cells include matrix metalloproteinase-7 (MMP7) and galactins (LGALS-3/LGALS-3BP/LGALS-9), which have been recently shown to be expressed in malignant cells (Crawford *et al.* 2002). These results support an association between CCC changes in the tumor microenvironment and PDAC progression.

### 4.3 PDAC LR survival analysis

To validate the LR predicted in the previous PDAC analysis, we performed survival analysis with LR pairs and individual genes by using bulk RNA-seq data (*n *=* *177) from the PAAD TCGA data (Raphael *et al.* 2017). We considered the top 10 positive (PDAC1) and negative (PDAC2) hits for the cell-pair (Malignant Ductal Cells, Ductal Cell) and performed survival analysis using the Cox Proportional-Hazards model (Andersen and Gill 1982), always keeping the stage information as a feature in each model.

For LR pairs with increased expression in PDAC2 cells (versus PDAC1), we observed 3 LRs with significant survival associations, where a higher LRScore leads to a worse prognosis. Two receptors also showed a significant signal (Supplementary Table S3). When comparing PDAC1 with PDAC2, we detected four significant LR interactions, four significant receptors, and two ligands (Supplementary Table S4). Also, in both cases, LR expression pairs displayed the highest significance (top 2 for PDAC2 and top 3 for PDAC1), which suggests that the composition expression of the predicted LR pairs is a better predictor of survival than individual genes. One example of a pair whose significance is higher than its individual ligand and receptors is MMP7 -> SDC1 (Supplementary Table S4). As previously mentioned, MMP7 has been associated with malignant ductal cells, and current research supports its role in early PDAC stages and tumor metastasis, leading to poorer prognosis (Van Doren 2022). Altogether, this analysis supports the translational potential of predictions by scACCorDiON.

### 4.4 Comparison with tensor-cell2cell

As an alternative to the previous analysis, we also explore using Tensor-cell2cell (Armingol *et al.* 2022), allowing a factor-based and sample-level interpretation. If we check the factors by comparing the two known class labels (Control versus PDAC), we observe that two factors (6 and 8) are significantly associated with controls; 5 factors (2, 3, 4, 5, and 7) with PDAC and one factor (1) is not related to the known labels (Supplementary Fig. S9A). Interestingly, by providing the clustering from scACCorDiON, we observe some factors to be related to PDAC1 (Factors 4 and 5) and others to PDAC 2 expression (Factors 1 and 7) (Supplementary Fig. S9B). These different loadings per sub-cluster support the biological relevance of these sample clusters.

Tensor-cell2cell can indicate cell–cell networks and LR pairs related to each factor for interpretation. Two factors (1 and 4) are related to the Malignant Ductal cell –> Ductal cell interaction (Supplementary Fig. S10A). Factor 1 is more prominent in PDAC 2 cells, and factor 4 is more prominent in PDAC 1 cells (Supplementary Fig. S9). Pathway analysis with progeny (Schubert *et al.* 2018) indicates that these factors are mostly related to similar pathways, such as TNFa, NFKb, and EGFR (Supplementary Fig. S10B). In contrast, the PDAC1 factor had higher activity for Hypoxia and TGFb pathways. An equivalent analysis of Ductal cell -> Ductal cell interactions predictions from scACCorDiON also finds similar pathway pathways except for a lack of TGFb signal (Supplementary Fig. S10C).

We next performed survival analysis equivalent to the one above by selecting the top 10 LR loadings for factor 1 and factor 4 (Supplementary Tables S5 and S6). To our surprise, only a single receptor pair, and four genes were predictors of survival compared to seven pairs and ten genes in scACCorDiON/CrossTalkeR predictions. A possible reason for this is that Tensor-cell2cell factors are not specific to cell–cell pairs or a sample group (PDAC 1 or PDAC 2), as the case for CrossTalkeR. Nevertheless, Tensor-cell2cell and CrossTalkeR can be seen as complementary and interpretable approaches, which can be used in a complementary manner.

## 5 Discussions and conclusion

scRNAseq-based LR analysis enables the inference of CCC events related to complex diseases. However, sample-specific analysis, crucial for understanding CCC events in patient cohorts, has only been addressed to a limited extent so far. Here, we explore the problem of clustering samples that share similar cell communication patterns by modeling sample-specific CCC as directed and weighted graphs. We propose a graph-based optimal transport framework that finds optimal probabilistic mappings between cell communication signals and cell–cell graphs. Furthermore, this framework allows us to measure the distance between any two directed weighted graphs (regarding a Wasserstein distance) and estimate “average directed weighted graphs” (barycenters) representing typical CCC patterns within a group of samples. Our algorithm is currently unique in that it allows both computing distances and clustering of directed weighted graphs. We have applied our DW-OT algorithm to calculate CCC graphs estimated in scRNA-seq with large cohorts and found that it outperforms other algorithms. An interesting result is that clustering worked better at fine resolution for datasets where coarse- and fine-level annotations were provided. This supports the idea that cellular sub-states are important in understanding cell–cell communication mechanisms.

In the DW-OT method, the “mass” of cell–cell communication signals is conserved between patients., i.e. it assumes that the same amount of cell–cell communication is present in both normal and disease samples. By utilizing prior biological knowledge, other works have explored mass variations in optimization problems via unbalanced Optimal Transport(UOT) (Peyré and Cuturi 2019), such as in cell development and proliferation (Schiebinger *et al.* 2019). There, prior knowledge is related to cell expansion or cell death, estimated from relevant pathway changes as supported by the expression profiles of the single cells. In our benchmarking, UOT did not improve our results. However, we lack a good source of prior biological knowledge of mass changes in cell–cell communication, as LR interaction follows complex Stoichiometry principles, the increase of a ligand does not mean more signal toward receptors due to saturation (Attie and Raines 1995). Modeling mass changes in cell–cell communication is an interesting venue for further research.

We further showcased how both barycenter and transport matrices can be used to interpret communication events supporting detected clusters. This usage is exemplified in the pancreas adenocarcinoma dataset, where DW-OT detected sub-clusters not characterized in the original study presenting the data (Peng *et al.* 2019). Using the signatures of the identified groups, we conducted a survival-based analysis using the TCGA-PAAD dataset on top LR pairs. Interestingly, LR expression was more significant than individual ligand and receptor genes. Moreover, we observed a tendency for higher enrichment in receptors than ligands, which potentially hints at the previously mentioned saturation aspect, i.e. an increase in ligands might not lead to an increase in cell–cell communication.

Future challenges include extending the DW-OT framework to work at the LR level. This implementation would require algorithms dealing with potentially large(nodes and/or edges) and noisy LR networks. We also noted that batch effects, frequently present in scRNA-seq cohort data, can affect sample level as analysis as well as of scRNA-seq data (Joodaki *et al.* 2024). Therefore, understanding and handling such effects opens a new venue for improvements in the current OT methods.

## Supplementary Material

btaf288_Supplementary_Data

## Data Availability

scACCorDiON code is available at https://github.com/CostaLab/scACCorDiON/ and https://scaccordion.readthedocs.io/en/latest/ and the survival analysis can be found at https://github.com/CostaLab/scACCorDiON.su. The data and results are available upon request at https://zenodo.org/doi/10.5281/zenodo.10808382.
