# Supplementary material for: scACCorDiON: a clustering approach for explainable patient level cell–cell communication graph analysis
Source: Bioinformatics. 2025 May 6;41(5):btaf288. doi: 10.1093/bioinformatics/btaf288 (PMC12122081; doi:10.1093/bioinformatics/btaf288)
Supplement: btaf288_Supplementary_Data [file btaf288_supplementary_data.pdf]

Supplementary Material

Supplementary Methods

Unbalanced Optimal Transport

The *unbalanced* optimal transport map $\Gamma^* \in \mathbb{R}^{E \times E}$ for two probability distributions defined on the nodes of the line graph as induced by the two CCC graphs $\mathcal{G}^k$ and $\mathcal{G}^l$ can now be computed as:

$$\Gamma^* = \arg\min_{\Gamma} \langle \Gamma, C \rangle_F + \epsilon \cdot \mathrm{KL}(\Gamma, C) + \lambda_1 \cdot \mathrm{KL}(\Gamma \mathbf{1} \| \mathbb{P}_{:,k}) + \lambda_2 \cdot \mathrm{KL}(\Gamma^T \mathbf{1} \| \mathbb{P}_{:,l}) \quad (1)$$

$$\text{where} \quad \mathcal{S} = \left\{ \Gamma \mid \Gamma \mathbf{1} = a, \Gamma^T \mathbf{1} = b, \Gamma_{ij} \geq 0 \right\},$$

and the associated (induced) Wasserstein distance between the two CCC samples is:

$$d_W(\mathcal{G}^k, \mathcal{G}^l) = \arg\min_{\Gamma} \langle \Gamma, C \rangle_F + \epsilon \cdot \mathrm{KL}(\Gamma, C) + \lambda_1 \cdot \mathrm{KL}(\Gamma \mathbf{1} \| a) + \lambda_2 \cdot \mathrm{KL}(\Gamma^T \mathbf{1} \| b) = \langle \Gamma^*, C \rangle_F \quad (2)$$

where the parameters λ_1 and λ_2 control the marginal relaxation of the transport plan Γ^*, row-wise(source) and column-wise(target), respectively; and the parameter ϵ controls the sparsity of Γ.

In contrast to the balanced OT formulation (Eq. **??**), this optimization requires three additional parameters. Higher λ_1 (λ_2) would impose a mass loss in the source (target) distributions. The ϵ is part of the sinkhorn optimization, which controls the sparsity of Γ, i.e. low values of ϵ result in a sparse transport plan Γ^*. For implementation details, we refer to POT documentation (`https://pythonot.github.io/gen_modules/ot.unbalanced.html#ot.unbalanced.sinkhorn_unbalanced2`).

Supplementary Tables

Method<br>Dataset	KBarycenter:CORR-OT	KBarycenter—DW-OT	Kmeans—Tabular	Kmedoids—CORR-OT	Kmedoids—DW-OT	Kmedoids—GOT	Kmedoids—Tabular	Leiden—CORR-OT	Leiden—DW-OT	Leiden—GOT	Leiden—Tabular
Kidney CKD/AKI	**0.303182**	0.134250	0.015940	0.087983	0.076468	-0.038452	0.018215	-0.014061	-0.017802	-0.023747	-0.057877
Breast Cancer	0.094386	0.000999	0.009027	0.115361	**0.276956**	0.067632	-0.007536	0.022934	0.018484	0.000000	0.018171
Pancreas Adenocarcinoma	-0.020883	0.034453	0.069089	-0.069666	**1.000000**	0.109974	0.066567	-0.034972	-0.034972	-0.030510	-0.046670
COVID	0.129534	0.001123	0.048706	0.147130	0.223579	**0.413028**	0.037638	0.023210	0.035998	0.014790	0.048582
Kidney Renal Carcinoma	**1.000000**	**1.000000**	-0.022337	**1.000000**	**1.000000**	-0.013245	-0.013245	0.114583	0.114583	-0.062500	0.060302
Lung Adenocarcinoma	**0.183582**	0.141784	-0.002409	0.134414	0.148882	0.048071	0.024293	-0.023132	-0.007003	0.000000	0.009701
Myocardial Infarction	0.141162	0.036802	-0.030983	0.250494	**0.713365**	0.624783	0.009031	-0.039031	0.165712	0.094465	0.062769

Table 1. Adjusted Random Index values for all clustering method and data sets. Best performing methods are highlighted in **Bold**.

	ARI	RI
RCC	**1.000000**	**1.000000**
RCC: Major Annotation	0.763889	0.882353
MI	**0.713365**	**0.861660**
MI: Major Annotation	0.434654	0.707510

Table 2. Results using two different cell type annotation granularity for Myocardial Infarction (MI) and renal clear carcinoma (RCC). These results are based on K-medoids using the DW-OT distances. Best performing annotation are highlighted in **Bold**

	LR model			Ligand model			Receptor model		
**Cluster**	**Terms**	**OR**	**p-value**	**Terms**	**OR**	**p-value**	**Terms**	**OR**	**p-value**
**PDAC2**	**SPP1_CD44**	1.547	0.004*	GRN	0.626	0.078	PLD2	0.458	0.017*
	GRN_SORT1	0.494	0.019*	GSTP1	1.441	0.169	**CD44**	1.467	0.024*
	GSTP1_TRAF2	1.851	0.037*	SPP1	1.092	0.203	MUC5AC	1.08	0.054
	ARF1_PLD2	0.458	0.106	CEACAM6	1.094	0.282	SORT1	0.77	0.202
	TFF1_MUC5AC	1.086	0.144	COL9A2	0.894	0.345	Stage II	1.619	0.28
	COL9A2_CD44	0.814	0.198	TFF2	0.957	0.484	CD4	0.936	0.593
	SLPI_CD4	0.826	0.301	MMP7	1.044	0.549	TRAF2	1.131	0.611
	TFF2_MUC6	0.954	0.35	Stage II	1.254	0.595	MUC6	1.021	0.613
	CEACAM6_CEACAM1	1.099	0.373	ARF1	0.887	0.771	Stage III+	1.135	0.863
	MMP7_ERBB4	0.958	0.52	TFF1	0.986	0.844	ERBB4	0.988	0.875
	Stage II	1.193	0.668	Stage III+	0.932	0.922	CEACAM1	1.015	0.895
	Stage III+	0.836	0.801	SLPI	1.007	0.945			

Table 3. Cox Proportional Hazards models results using the top 10 Malignant Ductal->Ductal cell related to the interaction related to PDAC2. (*) indicates statistically significant hits for survival prediction(p-value<0.05). Terms in **bold** indicate individual genes (ligand or receptor), which are also detected as significant as an LR pair.

	LR model			Ligand model			Receptor model		
Cluster	Terms	OR	p-value	Terms	OR	p-value	Terms	OR	p-value
**PDAC1**	C3_CD81	1.978	0.004*	**MMP7**	1.169	0.026*	ERBB3	1.428	0.022*
	**MMP7_SDC1**	1.408	0.005*	**TIMP1**	0.65	0.026*	RPSA	1.665	0.022*
	CD99_CD81	0.427	0.017*	C3	1.199	0.105	ITGB1	1.518	0.023*
	**TIMP1_FGFR2**	0.705	0.038*	LAMB2	0.706	0.168	CD63	0.562	0.032*
	S100A4_ERBB3	1.211	0.123	S100A4	1.108	0.186	SDC1	1.196	0.144
	TIMP1_CD63	0.704	0.16	CD99	0.787	0.283	CD81	0.805	0.23
	COPA_CD74	0.629	0.321	COPA	0.893	0.743	FGFR2	0.886	0.231
	LAMB2_RPSA	0.754	0.429	LGALS3BP	0.944	0.788	CD74	1.165	0.302
	Stage III+	0.681	0.6	Stage II	1.083	0.86	Stage III+	0.675	0.595
	APP_CD74	0.816	0.652	APP	1.027	0.922	Stage II	1.213	0.656
	Stage II	0.868	0.747	Stage III+	0.995	0.995			
	LGALS3BP_ITGB1	0.988	0.969						

Table 4. Cox Proportional Hazards models results using the top 10 Malignant Ductal->Ductal cell LR pairs related to PDAC1. (*) indicates statistically significant hits for survival prediction(p-value<0.05) and Terms in **bold** indicate individual genes (ligand or receptor), which are also detected as significant as an LR pair.

	LR model			Ligand model			Receptor model		
Cluster	Terms	OR	p-value	Terms	OR	p-value	Terms	OR	p-value
**Factor 4 (PDAC1)**	S100A4_ERBB3	1.67	0.08	HSPG2	0.707	0.036*	ERBB3	1.497	0.017*
	HSPG2_SDC1	0.611	0.103	APOE	0.841	0.042*	KCNQ1	0.814	0.056
	APOE_LSR	0.778	0.117	CALM1	0.634	0.089	SDC1	1.274	0.065
	FN1_SDC1	1.704	0.194	FN1	1.241	0.166	EGFR	1.33	0.096
	S100A4_ERBB2	0.688	0.275	HMGB1	0.749	0.386	LSR	0.863	0.384
	CALM1_KCNQ1	0.837	0.306	S100A4	1.063	0.421	Stage II	1.258	0.611
	COL3A1_DDR1	1.288	0.324	COL3A1	1.174	0.573	ERBB2	1.013	0.919
	HMGB1_SDC1	1.354	0.413	Stage II	1.178	0.702	DDR1	0.994	0.975
	Stage II	1.303	0.538	COL1A2	0.962	0.891	Stage III+	0.991	0.99
	S100A4_EGFR	0.983	0.947	Stage III+	0.995	0.994			
	Stage III+	0.969	0.965						
	COL1A2_SDC1	0.983	0.966						

Table 5. Cox Proportional Hazards models results using the top 10 LR pairs related Factor 4(PDAC1 high). (*) indicates statistically significant hits for survival prediction(p-value<0.05)

	LR model			Ligand model			Receptor model		
Cluster	Terms	OR	p-value	Terms	OR	p-value	Terms	OR	p-value
**Factor 1 (PDAC2)**	TFF3_CXCR4	0.819	0.031*	LAMC2	1.35	0.013*	PLSCR1	1.42	0.098
	CDH1_IGF1R	0.569	0.086	TFF3	0.953	0.329	CXCR4	0.856	0.192
	LAMC2_CD151	1.483	0.23	GDF15	1.055	0.463	CD44	1.262	0.192
	CDH1_PTPRF	1.36	0.279	Stage II	1.251	0.604	TGFBR2	1.213	0.342
	SLPI_PLSCR1	1.21	0.357	SPINT1	1.104	0.647	CD151	1.242	0.372
	SPINT1_ST14	0.798	0.483	CDH1	0.918	0.73	IGF1R	0.866	0.387
	LAMC2_CD44	1.204	0.53	MMP7	1.017	0.813	Stage II	1.302	0.546
	GDF15_TGFBR2	1.064	0.6	Stage III+	0.879	0.858	Stage III+	1.222	0.777
	Stage II	1.214	0.647	CGN	0.973	0.884	PTPRF	1.042	0.819
	MMP7_CD44	1.032	0.794	SLPI	1.012	0.899	ST14	1.005	0.978
	Stage III+	0.854	0.823						
	CGN_TGFBR2	0.945	0.835						

Table 6. Cox Proportional Hazards models results using the top 10 LR pairs related to Factor 1(PDAC2 high). (*) indicates statistically significant hits for survival prediction(p-value<0.05)

Supplementary Figures

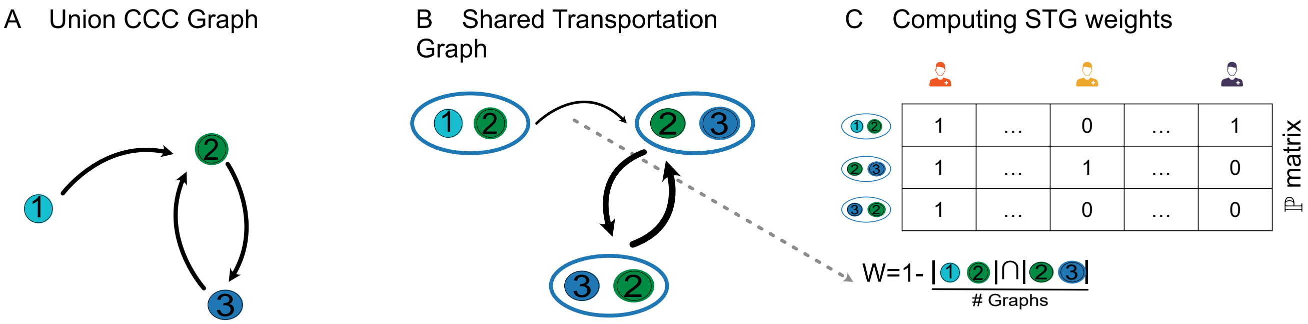

Suplementary Figure 1. Scheme for Shared Topology Graph: A) A union CCI graph is obtained by considering all directed interactions present in at least one sample CCI. B) The union graph is transformed to a line graph, where nodes are directed edges (1-¿2, 2-3, ...). C) The weights of the edges in the line graph in B) are the frequency at both interactions that appears in all sample-specific CCIs. The idea behind STD is that interactions that are more rare have a higher cost than common interactions.

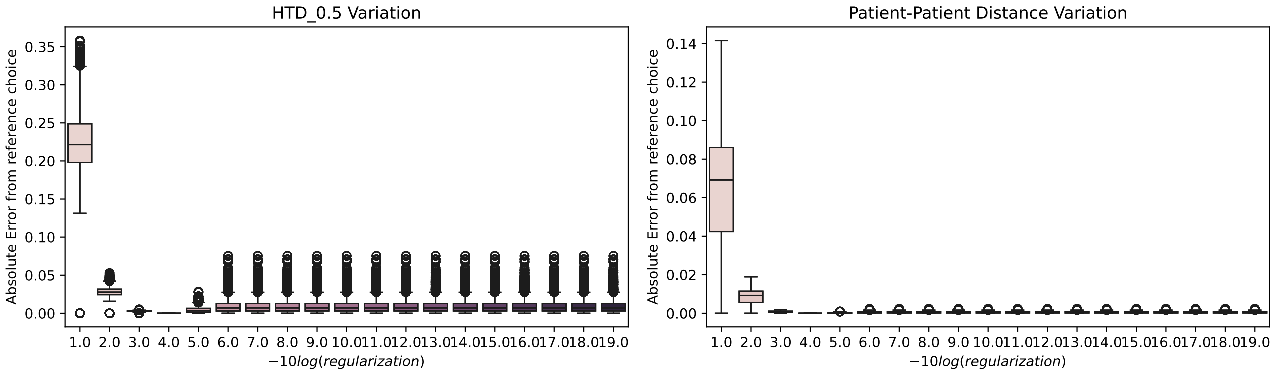

Suplementary Figure 2. Effect of the pagerank-like regularization effect in the PDAC dataset: A) Box plots show the differences/error (y-axis) between HTD cost matrix when distinct regularization terms (x-axis) are provided. B) Boxplot with the difference (distance) in the sample level distance matrix (y-axis) for distinct regularization terms (x-axis). In both cases, the errors are small for regularization terms smaller than 0.001.

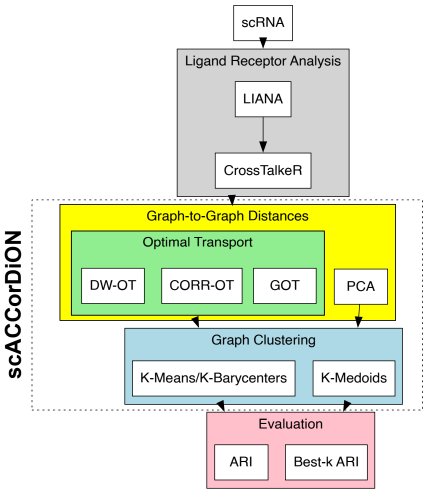

Suplementary Figure 3. Flowchart with the experimental design of this study and an overview of scACCorDiON. From the input scRNA cohort to the clustering benchmark

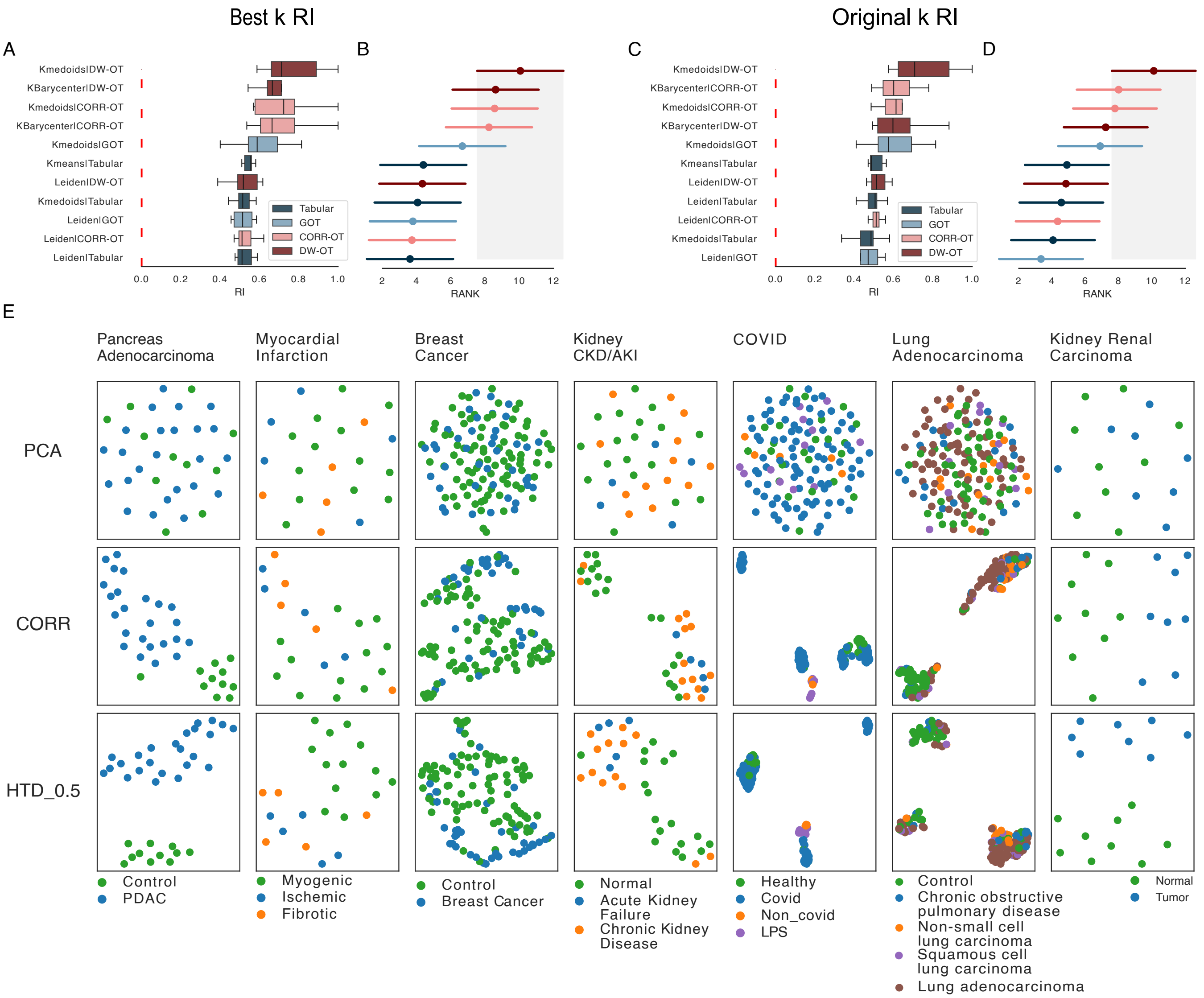

Suplementary Figure 4. Clustering Benchmark: A) Boxplots indicate the maximum RI value distribution (x-axis) distribution for all evaluated methods over five scRNA-seq data sets. B) Ranking values (mean and std) for each method and data set regarding the maximum RI value. The highest ranking indicates the highest RI. The gray area indicates the 95% confidence interval of the Friedman and Nemenyi posthoc-test). Methods whose average values are not within the gray area have significantly lower rankings than the top-ranked methods. For both A and B, methods are ranked by average in decreasing order. C and D are the same as A and B for the RI estimates, with the number of clusters equal to the number of original labels. E) UMAP two-dimensional embeddings of the distances matrices estimated by `Tabular`, `CORR-OT`, and `DW-OT` for all evaluated data sets. Colors correspond to the original labels.

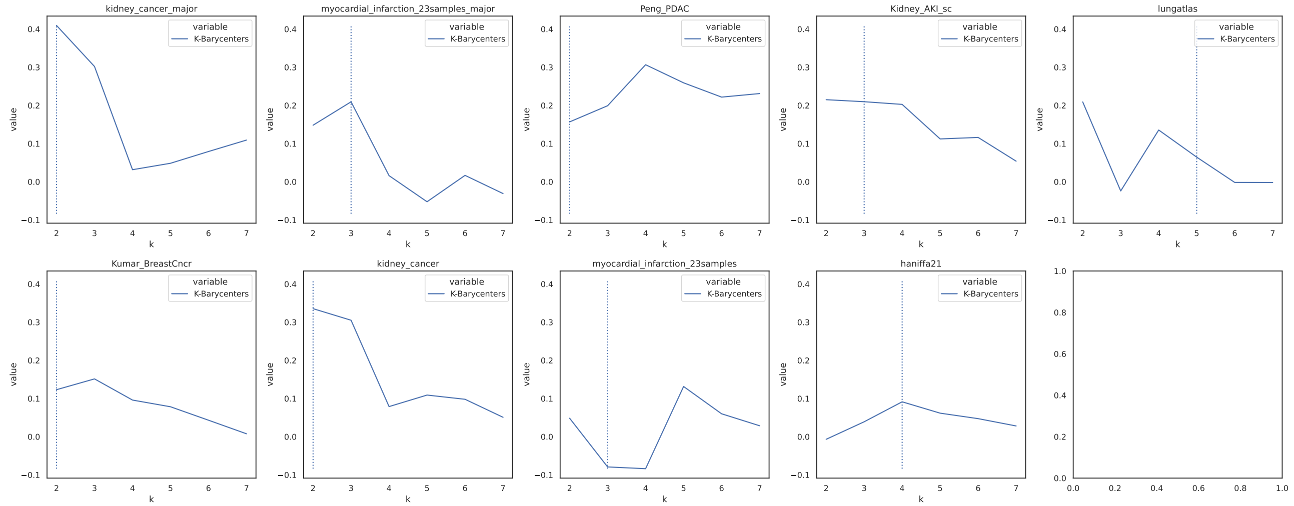

Suplementary Figure 5. Silhouette of different clustering methods using the DW-OT. Traced lines indicate the number of labels associated with each data set.

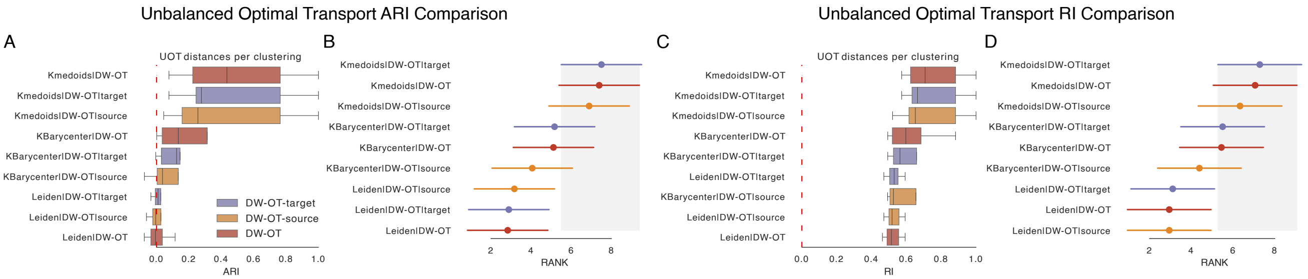

Suplementary Figure 6. A) Boxplots indicate the maximum RI value distribution (x-axis) distribution for all evaluated methods over five scRNA-seq data sets. B) Ranking values (mean and std) for each method and data set regarding the maximum ARI value. The highest ranking indicates the highest ARI. The gray area indicates the 95% confidence interval of the Friedman and Nemenyi posthoc-test). Methods whose average values are not within the gray area have significantly lower rankings than the top-ranked methods. For both A and B, methods are ranked by average in decreasing order. C and D is the same as A and B for the RI estimates, with the number of clusters equal to the number of labels. Here, we evaluated two versions of the unbalanced algorithm with an increase in mass in either the source ($\lambda_1 = 1, \lambda_2 = 50$) or target distribution ($\lambda_1 = 50, \lambda_2 = 1$). We observe that for k-medoids and k-barycenter, the balanced OT presents equivalent performance of all other approaches.

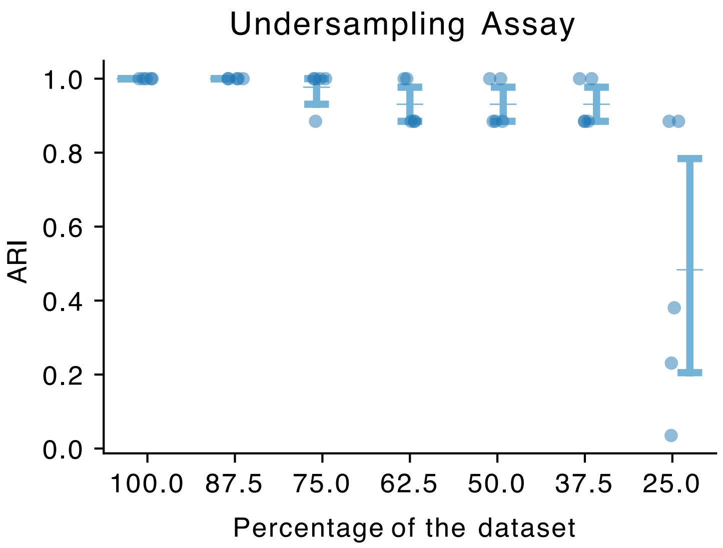

Suplementary Figure 7. Robustness to cell undersampling: ARI values (y-axis) vs. undersampling rates for the analysis of the PDAC data set with DW-OT k-medoids algorithms. To evaluate the robustness of scACCorDiON over the number of single cells in the data set, we under-sampled the PDAC dataset, keeping the following percentages of the total number of cells (25,37.5,50,62.5,75,87.5). For each of the percentages, we generated five replicates. Next, we re-run the LR analysis and performed the scACCorDiON k-medoids algorithm with DW-OT.

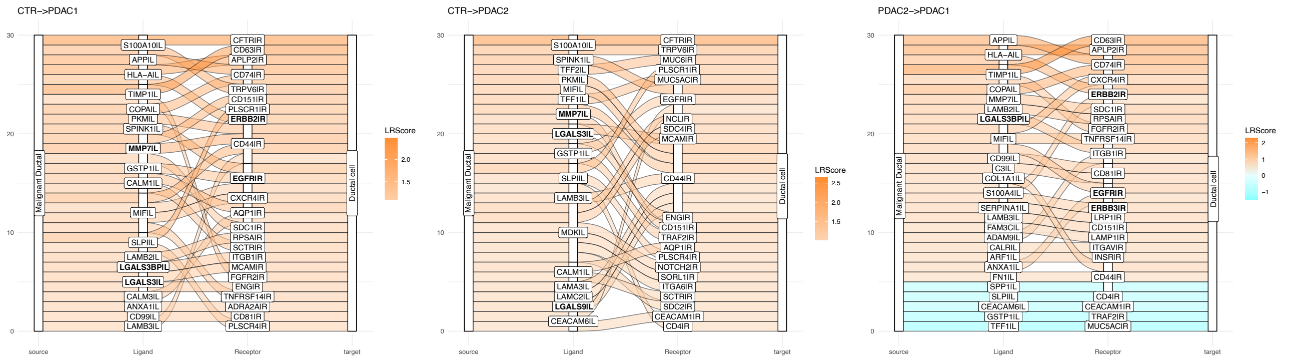

Suplementary Figure 8. Top 20 ligand-receptor pairs (highest absolute ligand-receptor expression values) between malignant ductal cells towards ductal cells between all three group pairs. We highlight LR pairs discussed in the text.

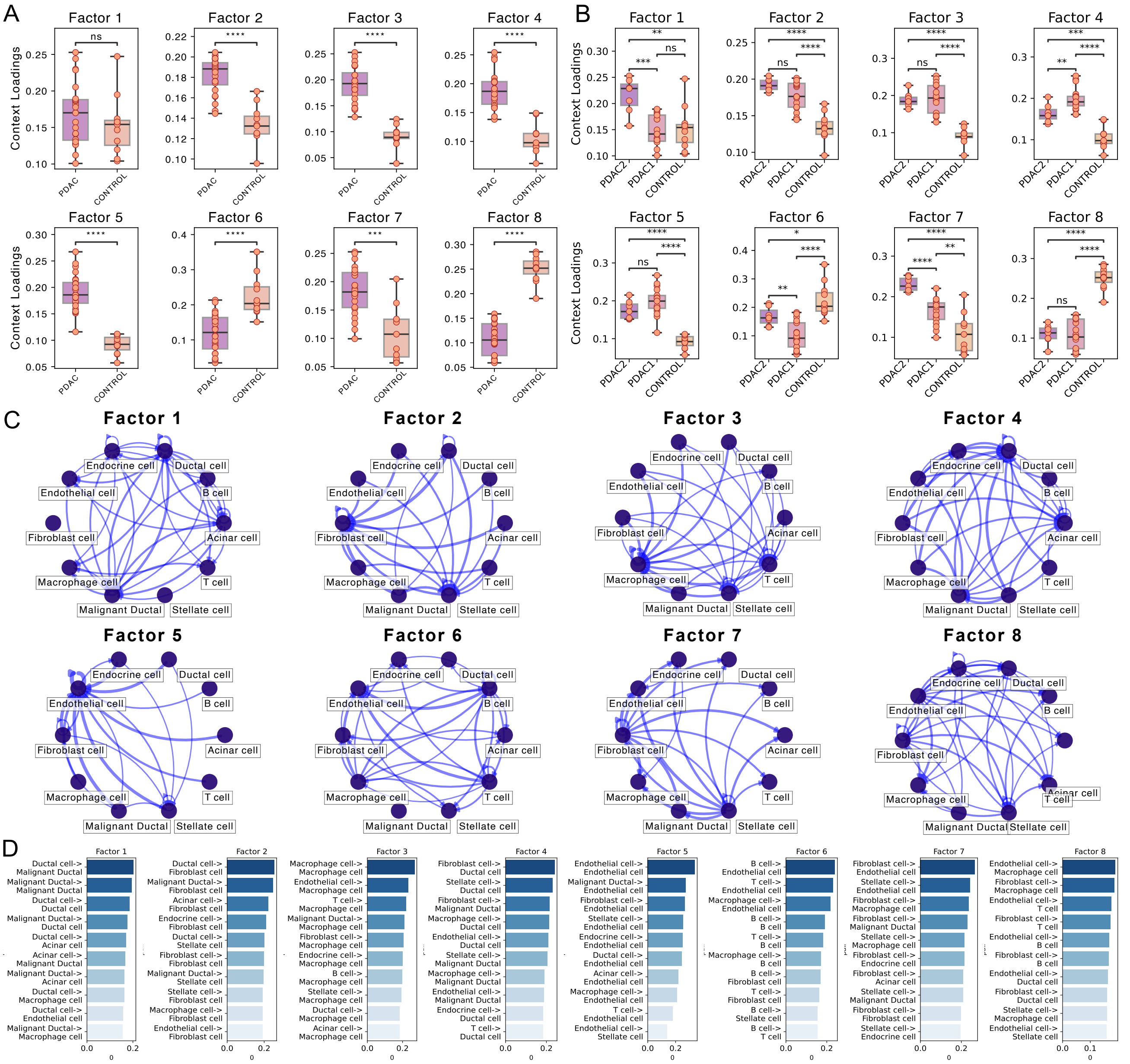

Suplementary Figure 9. Downstream analysis using Tensor-cell2cell: A) Statistical Comparison between the Context loading using the original disease labels; B) Statistical Comparison between the Context loading using the labels retrieved from scACCorDiON; C) Cell-Cell Communication Networks for each Factor. D) Top 10 highest cell-cell pairs' context loadings per factor.

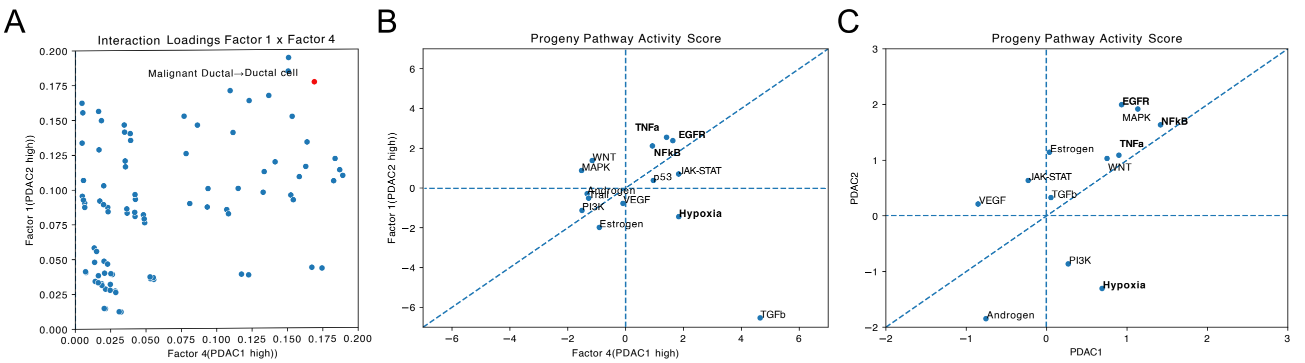

Suplementary Figure 10. Functional Analysis: A)Scatter plot with cell-¿cell interactions associated to PDAC2 related factor 1 (y-axis) and PDAC1 related factor 4 (x-axis). Both factors score the interaction Malignant Ductal cell -> Ductal Cells with high loading, which indicates its importance. B) Scatter plot with the progeny pathway activity for factor 1 (y-axis) vs factor 4 (x-axis). C) Scatter plot with progeny pathway activity for CrossTalkeR predicted PDAC 2 vs. PDAC 1 LR pairs for Malignant Ductal cell -> Ductal Cells.**Bold** indicates pathway activity found by both TensorCell2Cell and CrossTalkeR.
